# Epigenetic contribution to obesity

**DOI:** 10.1007/s00335-020-09835-3

**Published:** 2020-04-11

**Authors:** Meriem Ouni, Annette Schürmann

**Affiliations:** 1 Department of Experimental Diabetology, German Institute of Human Nutrition Potsdam-Rehbruecke (DIfE), Arthur-Scheunert-Allee 114-116, 14558 Nuthetal, Germany; 2 German Center for Diabetes Research (DZD), München-Neuherberg, Germany; 3 Institute of Nutritional Science, University of Potsdam, Potsdam, Germany

## Abstract

Obesity is a worldwide epidemic and contributes to global morbidity and mortality mediated via the development of nonalcoholic fatty liver disease (NAFLD), type 2 diabetes (T2D), cardiovascular (CVD) and other diseases. It is a consequence of an elevated caloric intake, a sedentary lifestyle and a genetic as well as an epigenetic predisposition. This review summarizes changes in DNA methylation and microRNAs identified in blood cells and different tissues in obese human and rodent models. It includes information on epigenetic alterations which occur in response to fat-enriched diets, exercise and metabolic surgery and discusses the potential of interventions to reverse epigenetic modifications.

## Introduction

Obesity is a complex disease caused by the interplay of genetics, epigenetics, and environmental factors. Different approaches such as linkage analysis, genome-wide association studies and exon as well as whole genome sequencing approaches identified numerous genes participating to the onset of severe obesity (Apalasamy and Mohamed 2015; Tam et al. 2019). Currently, more than 200 genetic loci are known to influence adiposity traits (Fall et al. 2017; Yengo et al. 2018). However, the heritability of obesity is not entirely attributable to gene variations, familial aggregation may also reflect epigenetic processes. In addition, epigenetic alterations occur in response to environmental exposures such by overnutrition (van Dijk et al. 2015; Weihrauch-Bluher et al. 2018) and not all changes are reversable.

Epigenetic mechanisms are defined as mitotically and/or meiotically heritable modulation of gene function without changes of DNA sequence (Wu and Morris 2001). Epigenetic modifications encompass DNA methylation, non-coding RNAs and histone modifications and are considered as an important source of inter-individual variability that might contribute to complex traits such obesity. DNA methylation is most frequently investigated in population-based studies, largely because of its relative stability and ease of measurement in high-throughput, array-based assays. In most cases DNA is methylated at the 5′ position of a cytosine residue, specifically at cytosine-phosphate-guanine dinucleotides (CpG) (He and Ecker 2015; Weber and Schubeler 2007). In mammals, this modification is known to play a role in cell fate and lineage. In somatic cells, DNA methylation patterns determined at defined developmental stages are faithfully maintained by DNA methyltransferases (DNMTs) (Weber and Schubeler 2007). Phenotypic plasticity in response to environment stimuli requires a flexible molecular machinery in each cell type. Therefore, the epigenome is susceptible to modifications during gestation, neonatal development, puberty, and aging. When epigenetic patterning is established at specific genomic loci before gastrulation, it results in systematic interindividual variation within one cell type; it is designated metastable epiallele. Metastable epialleles are alleles that are variably expressed in genetically identical individuals due to epigenetic modifications that take place during early development; they are thought to be particularly vulnerable to environmental influences (Dolinoy et al. 2007; Finer et al. 2011). Other loci can also be epigenetically affected in response to environmental exposures; however, these changes occur in a tissue-specific manner and in adulthood (Jirtle and Skinner 2007; Saussenthaler et al. 2019).

MicroRNAs (miRNAs) are small non-coding RNAs of 21 to 25 nucleotides which act as crucial post-transcriptional regulators of gene expression. They bind to cis-elements in the 3′ untranslated region (3′UTR) of mRNAs and act as silencer because they induce the degradation, impair the stability or inhibit translation of their targets (Bartel 2004; Guo et al. 2010). Most miRNAs bind to more than one target mRNA and multiple miRNAs can cooperate to fine-tune the expression of one single transcript (Doench and Sharp 2004; Grimson et al. 2007; Selbach et al. 2008).

The review presents major epigenetic alterations on the level of DNA methylation and changes of miRNA observed in relation to obesity. In most cases we will first refer to human data followed by results obtained in rodent.

## Impact of intrauterine and early-life environment on DNA methylation in obesity

**Fig. 1:**
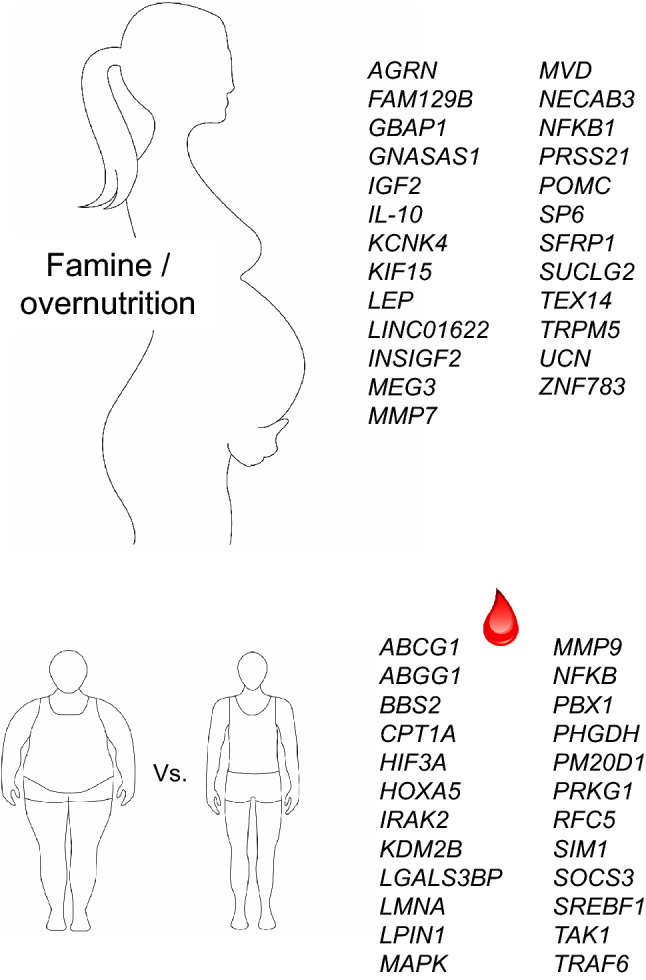
Epigenetic changes in obesity. Changes in DNA methylation in utero in response to under- and overnutrition (upper panel) and in adulthood as detected in blood cells (lower panel)

A variety of epidemiological observations provide evidence that adult-onset diseases are linked to in-utero exposures. Both extremes, fetal under- and overnutrition, which are regulated by maternal diet, are associated with an increased risk of obesity and the metabolic syndrome (Fernandez-Twinn et al. 2019; Harder et al. 2007; Heijmans et al. 2008). Two prominent examples, the Dutch Hunger Winter in 1944–1945 (Kyle and Pichard 2006) and the Chinese famine in 1959–1961 (Li and Lumey 2017) showed that individuals prenatally exposed to famine were more prone to be overweight and to develop obesity-associated diseases like T2D and CVD than those born years before. There are several indications that epigenetic changes during early development mediate the outcome in later life. A lower degree of DNA methylation of the imprinted *IGF2* gene was detected in blood cells of people born during the Dutch Hunger Winter compared with same-sex siblings that were unexposed. As the association was specific for periconceptional exposure it was concluded that very early mammalian development is a crucial period for establishing and maintaining epigenetic marks (Heijmans et al. 2008). Later, several other genes including *INSIGF2*, *GNASAS1, MEG3, IL-10*, and *LEP*, some of which have a known role in metabolic disorders, were identified to exhibit an altered DNA methylation in blood cells of the offspring of famine-exposed mothers (Tobi et al. 2009) (Fig. 1, upper panel).

However, nowadays most populations are confronted with an obesogenic environment and prenatal and neonatal overfeeding programs a permanent obesity disposition. Earlier studies on effects of intrauterine effects on DNA methylation were focused on candidate genes. For example, epigenetic alterations in the *Pomc* gene, known for its important role in the regulation of food intake, were first observed in rats and later confirmed in human. Neonatal overfeeding of rats led to rapid early weight gain and the metabolic syndrome, which was associated with a hypermethylation of the *Pomc* promoter and the inability of a *Pomc* upregulation in response to elevated leptin and insulin concentrations (Plagemann et al. 2009). Accordingly, in postmortem human melanocyte-stimulating hormone (MSH)-positive neurons’ alterations in DNA methylation of the *POMC* gene were strongly associated with individual body mass index (BMI). Epigenetic alterations of *POMC* appeared to be established in the early embryo and offspring methylation correlated with the paternal somatic methylation pattern (Kuhnen et al. 2016).

Later studies were based on well-characterized cohorts, for example, the Avon Longitudinal Study of Parents and Children (ALSPAC) cohort, in which in 88 participants an elevated gestational weight gain in early pregnancy (0–18 weeks of gestation) was associated with increased DNA methylation of four CpG sites (*MMP7, KCNK4, TRPM5, NFKB1*) in offspring cord blood DNA (Morales et al. 2014). In 1018 participants of the same cohort, 28 CpG sites (e.g., in *SUCLG2, FAM129B* and *KIF15*) were differentially methylated in the offspring of obese and 1621 *(*e.g., in *TEX14*, *ZNF783, MVD*) of underweight mothers, in comparison to the offspring of normal weight mothers. The fact that the associations of maternal obesity with offspring methylation were stronger than associations of paternal obesity supports the intrauterine influence (Sharp et al. 2015).

In contrast a meta-analysis of 19 cohorts with close to 10,000 mother–newborn pairs provided evidence that epigenetics plays a minor role for the association of an elevated body weight of the mother during early pregnancy with the overweight of her child. After adjusting for cell proportions, 86 sites showed changes in DNA methylation in newborns with an association of maternal BMI at the start of pregnancy. However, including results of an independent cohort of adolescents left only 8 sites (in or near *SP6, NECAB3, AGRN, SFRP1, GBAP1, LINC01622, UCN, PRSS21*) supporting evidence for a causal intrauterine effect (Fig. 1, upper panel). This led to the conclusion that genetic and/or lifestyle factors play a more important role for intrauterine mechanisms than changes in DNA methylation (Sharp et al. 2017).

## Changes in DNA methylation associated with obesity in human

Changes in DNA methylation are dynamic and prone to be affected by environmental factors during early development. However, although less dynamic, epigenetic modifications also take place throughout postnatal life and adulthood. About one-third of all methylated sites in blood cells change age-dependently (Ronn et al. 2015). Screenings for epigenetic alterations in humans are mainly performed in blood cells, in particular when larger cohorts are investigated in epigenome-wide association studies (EWAS). There is a constant increase in studies described in the literature which performed array-based EWAS and we will try to focus on the most relevant ones, meaning those (i) performed with cohorts of more than 1000 participants, or (ii) studies which include data of at least two different time points or (iii) in which beside blood cells also a tissue was analyzed. An important and early study by Feinberg et al. (2010) analyzed 4.5 million CpG sites in 74 individuals at two time points, about 11 years apart. The authors detected more than 200 variably methylated regions (VMRs) distributed over the entire genome. Half of these VMRs were stable within individuals over the time and defined as personalized epigenomic signature. Four VMRs showed covariation with BMI consistently at both study visits and were located in or near genes previously implicated in regulating body weight or diabetes such as the metalloproteinases *PM20D1* and *MMP9* that were earlier described to be upregulated in obese individuals, or *PRKG1*, a protein kinase involved in the regulation of energy balance, and *RFC5* involved in DNA repair (Feinberg et al. 2010). These results clearly support the idea to use epigenetic marks for stratifying patients at risk of common diseases (see below). EWAS was performed in leukocytes obtained from 2097 African American adults in the Atherosclerosis Risk in Communities (ARIC) study and replication achieved by analyzing two additional cohorts (Demerath et al. 2015). Changes in DNA methylation were associate to specific obesity-related traits, revealing 76 BMI-related sites, 164 waist circumference-related and 8 BMI change-related sites which included already reported ones (*HIF3A, CPT1A, ABCG1*) and new candidates (e.g., *LGALS3BP, KDM2B, PBX1, BBS2*) (Fig. 1, lower panel) which were also affected in adipose tissue and involved in lipid metabolism and immune response/cytokine signaling (Demerath et al. 2015). Another study with 5,387 individuals from three different cohorts (two European, EPICOR, KORA and one Indian Asian, LOLIPOP) identified 187 genetic loci with an altered DNA methylation which associated with elevated BMI (Wahl et al. 2017). A weighted genetic risk score (GRS) and Mendelian randomization demonstrated that most of these epigenetic alterations were predominantly the consequence of adiposity, rather than the cause. The methylation loci were linked to genes involved in lipid and lipoprotein metabolism, substrate transport and inflammatory pathways (e.g., *ABGG1, LPIN1, HOXA5, LMNA, CPT1A, SOCS3, SREBF1, PHGDH, NFKB, MAPK, TAK1, IRAK2 and TRAF6*) (Fig. 1, lower panel) and that disturbances in DNA methylation predict development of obesity-related disorders like T2D.

An important question is how stable epigenetic alterations are. Comparisons of human data of adults with those of children and adolescents as well as time course studies are not described so far. Therefore, we evaluated results of a large cohort of adults (Wahl et al. 2017) with two studies in which 117 (Fradin et al. 2017) and 702 (Huang et al. 2015) differentially methylated genes were observe to be associated with childhood obesity. Among these candidates only 5 and 19 genes, respectively, showed changes in DNA methylation in both children and adults (Table 1), indicating that (i) most epigenetic alterations are not stable, or (ii) that the overall heterogeneity of study participants is too large to detect stable changes.

**Table1:** Overlapping genes affected by changes in DNA methylation in obese children (Fradin et al. 2017; Huang et al. 2015) and obese adults (Wahl et al. 2017)

Huang et al. (2015)	Fradin et al. (2017)
*ABCC5*	
*ARID1B*	
*CD247*	
*CHD3*	
*CNTN1*	
*CPNE6*	
*EEFSEC*	
*FAM53B*	
*GABBR1*	
*IGFBP6*	
*KCNQ1*	
*MAD1L1*	*MAD1L1*
*RPS6KA2*	
*SBNO2*	
*SH2B2*	
*SLC43A1*	
*SLCO3A1*	
*STK40*	
*SYNJ2*	
	*CBFA2T3*
	*CPT1A*
	*HDAC4*
	*P4HB*

Results of animal studies indicated that several epigenetic alterations visible early in life are stable. We have used genetically identical mice which differ in their response to high-fat diet (HFD) feeding. In livers of 6-week-old mice which are prone to become obese, we detected an elevated DNA methylation and lower expression of *Igfbp2* (Kammel et al. 2016) and a lower methylation and higher expression of *Dpp4* (Baumeier et al. 2017a) than in mice that were protected from diet-induced obesity. Both changes were stable and detectable in adult mice at the age of 22 weeks (Baumeier et al. 2017a; Kammel et al. 2016). Interestingly, both genes have a functional impact on the disease and were also affected in human (Ahrens et al. 2013; Baumeier et al. 2017a, b). For *IGFBP2*, we even observed a prospective association of its circulating concentration and of its differential DNA methylation with T2D incidence (Wittenbecher et al. 2019). These findings support the advantages of animal studies for translational approaches.

As older people are more prone to develop obesity, the question arises to what extent epigenetic changes associated with obesity are age-related and therefore not directly related to obesity. According to actual data, it is difficult to distinguish between both parameters because obesity-related epigenetic changes have not been compared to age-related changes. In one recent study, Salas-Perez et al. (2019) aimed to understanding the implication of the epigenome on the development of age-related metabolic alterations. Close to 500 participants were categorized according to age (< 45- vs > 45-year old) and the presence of metabolic diseases including obesity. In leukocytes of young and older people more the 13,000 CpG sites showed significant methylation differences, of which 58 were located in genes linked to longevity-regulating pathways and strongly correlated with obesity. These include *MTOR, INS, ULK1, ADCY5, ADCY6, CREB5, IGF1R, RELA* and *PPKAG2* which are related to waist circumference and statistically associated with age. Several of them showed also an association between altered DNA methylation and obesity in other studies. However, up to now little is known about the trajectories of epigenetic changes and which period of the adult life is particularly sensitive for epigenetic alterations that affect body weight, metabolic diseases and longevity.

## Epigenetic plasticity: influence of interventions on epigenetic alterations

As stated above, epigenetic alterations are dynamic and might be corrected through targeted interventions. The most important and successful interventions to decrease body weight and improve the metabolism are nutritional changes, exercise and metabolic surgery. In this chapter, we will summarize relevant effects on DNA methylation that were induced by the listed treatments.

### Effects of diets on DNA methylation

The role of nutrition on epigenetics during early development represents a period in which epigenetic errors may have major consequences for the health. However, we will now discuss which epigenetic alterations occur later in life via a prolonged unhealthy diet.

In particular diets with an enriched fat content lead to elevated body weight and ectopic fat storage (e.g., in the liver), which both have negative effects on insulin sensitivity. The group of C. Ling studied the impact of 7-week-lasting intake of extra amounts (+ 750 kcal/day) of saturated (SFA) or polyunsaturated fatty acids (PUFA) on the genome-wide DNA methylation in subcutaneous adipose tissue (SAT) of young healthy humans. Both interventions did not differ in their degree of body weight gain but in the degree of DNA methylation of adipose tissue. Comparing both diets revealed 1797 genes with a differential methylation in response to PUFA (e.g., *POMC, FTO, IL6, INSR, NEGR1*) and 125 genes (e.g., *ADIPOQ*) after SFA overfeeding. However, expression changes were only found for 28 mRNAs (e.g., *ACOX1, FAT1*) in SAT, not in the PUFA group (Perfilyev et al. 2017). Effects of a short-term high-fat overfeeding on genome-wide DNA methylation patterns were analyzed in human skeletal muscle biopsies from 21 healthy young men in a randomized crossover setting. HFD induced DNA methylation changes in 6,508 genes. Among the top 20 most significant genes were *DNM2, MGMT, GLUT3, MRC1* and *ACAT2.* After diet switch, only a trend toward reversibility of the DNA methylation was found in skeletal muscle of 10 participants (Jacobsen et al. 2012). Brøns and colleagues studied the effect of a 5-day fat-enriched diet (+ 50% calories) on the epigenome of the skeletal muscle and detected elevated methylation and decreased gene expression of *PGC-1α* and two OXPHOS genes in subjects with low birth weight, not in those with a normal birth weight (Brons et al. 2010). Focusing on subjects with low birth weight, who are prone to become obese and insulin resistant, 5-day HFD resulted in upregulation of *ELOVL6, FADS2* and *NNAT* and downregulation of *INSR, IRS2* and the fatty acid transporter *SLC27A2* in SAT. Changes in DNA methylation were detected at 652 CpG sites (including in *CDK5, IGFBP5* and *GLUT4*) but did not show an overlap to expression changes (Gillberg et al. 2016). This indicates that a short-term intervention of less than 1 week has only limited effects on DNA methylation changes which affect gene expression.

### Effects of caloric restriction on DNA methylation

**Fig. 2:**
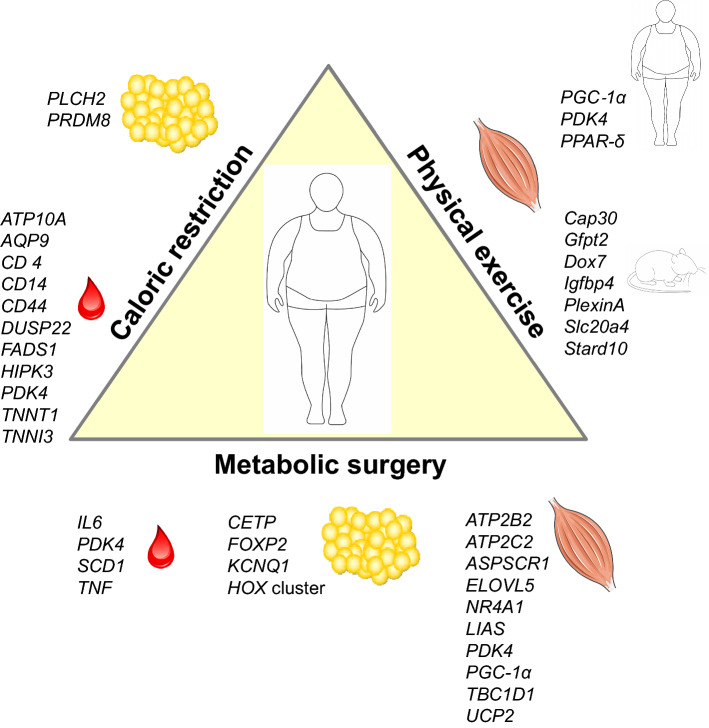
DNA methylation plasticity. Summary of genes exhibiting alterations of DNA methylation in response to the indicated interventions for treatment of obesity. Genes and tissues affected by DNA methylation in the indicated interventions are mentioned. The lists are not complete, but show the candidates mentioned in the text of this review

Reducing caloric intake by adjusting meal size or meal frequency (time-restricted feeding, intermittent fasting) has beneficial effects on health, such as reducing body weight and delaying the onset of chronic diseases like T2D (Di Francesco et al. 2018; Parrillo et al. 2019). An elegant study was performed by Milagro et al., evaluating DNA methylation pattern in PBMCs before and after a short-term (8 weeks) caloric restriction. Hypocaloric diets changed DNA methylation at several sites of *ATP10A* and *WT1.* Interestingly, the methylation patterns of *ATP10A, AQP9, CD44 DUSP22, HIPK3, TNNT1*, and *TNNI3* could be used as early indicators of the intervention success, indicating that the interindividual variability of the epigenetic background plays a role on the effectiveness of a weight management program (Milagro et al. 2011; Moleres et al. 2013). Another short-term caloric restriction in overweight women was shown to decrease methylation of *CD36, CD14, PDK4*, and *FADS1* in PBMCs (do Amaral et al. 2014; Hernandez-Saavedra et al. 2019). A long-term caloric restriction (6 months) with a 4-week weight maintenance period resulted in more than 3% reduction of body fat and in hypermethylation of *PLCH2* and *PRDM8* in SAT biopsies (Bouchard et al. 2010) (Fig. 2). Overall, it appears that methylation changes occur more easily by overfeeding than been reversed by reducing caloric intake (Ling and Ronn 2019).

### Exercise and DNA methylation

The adaptive response of skeletal muscle to exercise training is tightly controlled. It requires transcriptional regulation and changes in DNA methylation. Acute high‐intensity exercise resulted in a hypomethylation and elevated expression of *PGC‐1α, PDK4*, and *PPAR-δ* in human and mice (Barres et al. 2012). A broader screen in mice was performed by us. A genome-wide analysis of DNA methylation in muscle of trained mice (5 days/week for 4 weeks) identified more the 2700 genes with significant methylation changes in their putative promoter regions compared with sedentary controls. Two hundred genes exhibiting an altered DNA methylation and expression were related to muscle growth and differentiation (e.g., *PlexinA, Igfbp4, Dox7*), a minor fraction was linked to metabolic regulation (e.g., *Gfpt2, Cap30, Stard10, Slc20a4*) (Kanzleiter et al. 2015) (Fig. 2).

To evaluate whether epigenetic changes occurring after exercise can reverse those induced by a fat-enriched diet, the group of R. Barres investigated the transcriptome and methylome of human skeletal muscle before and after 9 days of HFD with or without three bouts of resistance exercise training in middle-aged, sedentary males. The 9-day HFD resulted in expression changes of about 400 genes of which only 10 revealed alterations in DNA methylation. Six times more genes were affected when HFD was combined with exercise; however, among these only 54 were affected by DNA methylation, clearly indicating that the latter plays a minor role for positive effects of short-term training (Laker et al. 2017).

### Changes of DNA methylation induced by metabolic surgery

It is well accepted that metabolic surgery is the most effective strategy to treat obesity and its associated comorbidities (Hao et al. 2016; Schauer et al. 2017). The review by Nicoletti et al. summarizes genes which have been identified to be epigenetically altered after metabolic surgery. Most changes (*IL6, PDK4, TNF, SCD1*) were detected in whole blood cells about 12 months after the surgery. In adipose tissue, the surgery changed DNA methylation of *CETP, FOXP2, KCNQ1*, *HDAC4 DNMT3B* and *HOX* clusters (Fig. 2) (Nicoletti et al. 2017). Similar to the effects induced by exercise, *PGC-1α* and *PDK4* were affected in skeletal muscle after the operation. We investigated the effects of metabolic surgery on epigenetic, transcriptional and metabolic changes in skeletal muscle and their dynamic temporal relationships during the improvement of insulin sensitivity between 2 weeks and 12 months after metabolic surgery. The surgery-mediated weight loss was not immediately followed by improved muscle insulin sensitivity, likely related to transiently augmented lipolysis resulting in accumulation of lipid intermediates, inadequate mitochondrial function and altered gene expression profiles. After 2 weeks, nearly no changes in DNA methylation occurred. At 12 months, we detected effects on DNA methylation at 1467 CpG sites located in/or close to regions of 430 differentially expressed genes. They are enriched in glucose homeostasis (*TBC1D1*, *NR4A1*, *ASPSCR1*), mitochondrial function (*LIAS*, *UCP2*), lipid metabolism (*ELOVL6*) and calcium signaling (*ATP2B2*, *ATP2C2*) (Gancheva et al. 2019) (Fig. 2). Thus, several beneficial effects observed 1 year after the metabolic surgery on metabolism, immunity and inflammation appeared to be mediated by epigenetic mechanisms (Izquierdo and Crujeiras 2019). However, more longitudinal and large studies are needed because up to now only small cohorts were characterized. In addition, it is still not clear if epigenetic marks are sufficient to predict a long-term success of the surgery.

## Altered miRNAs in obesity

As each miRNA supresses several targets and miRNAs can cooperate to inhibit specific genes, they play key roles in numerous physiological processes, including cell proliferation, apoptosis and cell identity, but also in pathological processes such as obesity and the metabolic syndrome (Nicoletti et al. 2017; Treiber et al. 2019).

## miRNAs involved in the regulation of adipocyte differentiation and adipose tissue function

**Fig. 3:**
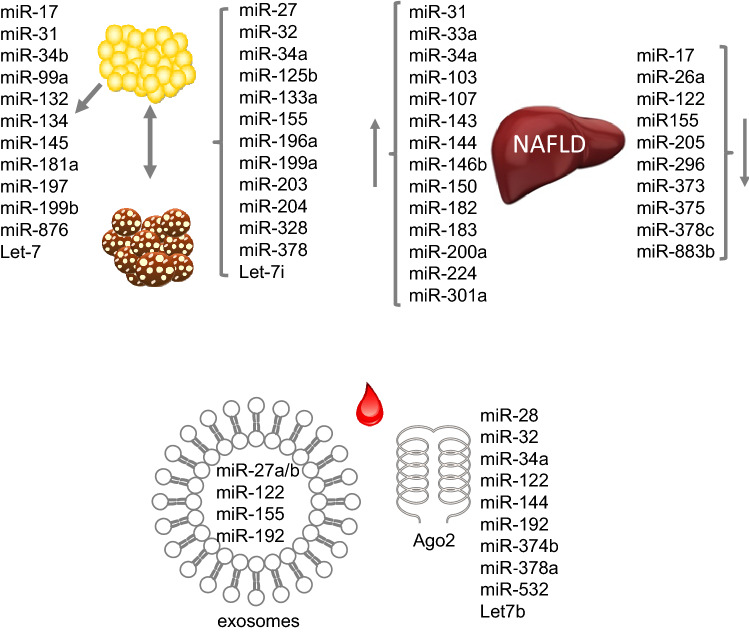
miRNA in obesity. miRNAs with an altered level in white and brown adipose tissue or liver in the state of NAFLD (upper panel). miRNAs detected in plasma either in exosomes or bound to the protein Ago2 (lower panel). The lists are not complete, but show the candidates mentioned in the text of this review

Adipose tissue is one of the most affected tissues in the state of obesity and miRNAs affect adipocyte differentiation, lipogenesis and insulin sensitivity and thereby participate in the development of obesity. The evaluation of miRNA levels during differentiation process revealed a downregulation of miR-34b, -197, and -199b during the differentiation of 3T3-L1 adipocyte. Likewise, miR-31 which was detected in an obesity locus is suppressed during differentiation of human adipocytes, the SGBS cell line. Our results showed that elevated levels of miR-31 inhibited the expression of *PPP2r5a*, *PPARy*, *IRS1*, and *GLUT4* (Gottmann et al. 2018)*.* MiR-27a and -27b impair human adipocyte differentiation by targeting *PPARγ*, *LPL* and *C/EBPα*. MiR-378/378* seems to be important for efficient lipid uptake and hypertrophy of adipocytes. The Let-7 family was shown to play an important role in adipocyte differentiation by targeting *HMGA2* encoding a protein involved in the regulation of the cell cycle and the transition from clonal expansion to terminal differentiation. However, it was shown that overexpression of Let-7 in adipose tissue of mice did not change the fat mass (Frost and Olson 2011). Beside affecting differentiation processes, miRNAs alter adipocyte function. Our own in vitro experiments suggested that *GLUT4*, *IRS1*, key genes in insulin signaling pathways, are targeted by miR-31 (Gottmann et al. 2018). Another example is miR-876 which regulates glucose homeostasis; its dysregulation resulted in insulin resistance because miR-876 binds to 3′UTR of adiponectin and reduces the levels of this insulin sensitizing cytokine (Rajan et al. 2018). In humans, there are significant correlations between the expression of miR-17, -99a, -132, -134, -145, -181a, -197 (Fig. 3). And adipose tissue morphology and key metabolic parameters (e.g., HbA1c, fasting plasma glucose, and circulating leptin, adiponectin, interleukin-6) were identified (Kloting et al. 2009).

## Role of miRNAs for the development of brown and beige adipocytes

MiRNAs also play a role in brown adipose tissue (BAT) as well as in beiging/browning processes in humans and rodents. As BAT dissipates energy as heat via thermogenesis, its activation increases energy expenditure and might protect against obesity (Thyagarajan and Foster 2017). The brown transcriptional network which includes the gene products of *Prdm16, Pparα, Creb*, and *Pgc1β* (Mori et al. 2012) is targeted by multiple miRNAs, miR-27 (Sun and Trajkovski 2014), -32 (Ng et al. 2017), -34a (Fu et al. 2014), -328 (Oliverio et al. 2016), and -378 (Pan et al. 2014). The amount of BAT decreases during aging and obesity development. Not only the loss of brown adipocytes but also the beiging of WAT might be mediated—at least partially by miRNAs. For example, miR-133a and miR-155 inhibit formation of BAT and enhance transition of WAT to a beige phenotype (Chen et al. 2013; Liu et al. 2013). In contrast, miR-203 promotes WAT browning in cold-exposed mice and improves glucose tolerance in HFD-fed mice by silencing *Lyn* and thereby repressing IFN-γ. miR-378 suppresses the expression of phosphodiesterase *Pde-1b* in BAT but not in WAT, and miR-378 and *Pde1b* inversely regulate brown adipogenesis in vitro (Pan et al. 2014). Furthermore, miR-196a inhibits the expression of the WAT gene *Hoxc8* during the brown adipogenesis. Expression of miR-196a is induced in WAT-progenitor cells of mice in response to cold exposure or β-adrenergic stimulation. Transgenic mice overexpressing miR-196a in adipocytes are protected from obesity due to an increased energy expenditure. Thus, miR-196a participates in the development of metabolically functional brown-like cells (Mori et al. 2012). miR-32 is specifically expressed in BAT and upregulated in response to cold exposure. Accordingly, cold tolerance was impaired by suppression of this miRNA. Another interesting regulator is miR-199a, because its expression decreases during the differentiation of brown adipocytes and at the same time increases during white adipocyte differentiation in mice and humans. Thermogenic activation suppresses miR-199a expression, whereas it increases during aging. *mTOR* is an important regulator of brown adipogenesis and thermogenesis and a direct target of miR-199a (Gao et al. 2018). In comparison to WAT expression of miR-125b is lower in BAT in mice and humans. Its overexpression decreased mitochondrial biogenesis while its knockdown had opposite effects, indicating that miR-125b is a suppressor of WAT beiging (Giroud et al. 2016b). In a screen performed in human adipose tissue Let-7i was found to be lower expressed in beige adipocytes. The overexpression of Let-7i mimic in human beige adipocytes decreased *UCP-1* expression (Giroud et al. 2016a). A recent mouse study demonstrated an upregulation of miR-204 in the fetal BAT of obese mothers. miR-204 targets *Pgc-1α* and *Sirt1* mRNA which are both essential regulators of mitochondriogenesis and might explain how maternal obesity predisposes offspring to metabolic diseases (Wang et al. 2019). Presumably due to the fact that the amount of BAT in human is small and difficult to collect, no studies are described on the role and expression profiles of miRNAs in this tissue in humans (Fig. 3).

## Role of miRNAs in the liver of obese individuals

Most obese subjects develop a non-alcoholic fatty liver disease (NAFLD), which leads to hepatic insulin resistance. Changes in the expression of miRNAs appear to be involved in impaired liver metabolism. The review by Torres et al. summarized those miRNAs which were affected in livers from patients with NAFLD and compared those with the status of alcoholic liver disease (ALD). miR-31, -33a, -34a, -144, -146b, -150, -182, -183, -200a -224, and -301a were higher abundant and miR-17, -122, -296, -373, -375 and -378c lower expressed in NAFLD patients. Among these, miR-34a, -122, and -155 have been associated with the pathogenesis of NAFLD and affected in ALD (Torres et al. 2018) (Fig. 3). miR-34, which is elevated in fatty liver of mice and humans and also detected in plasma, targets *SIRT*, an important regulator of energy homeostasis (Castro et al. 2013; Wu et al. 2014). miR-122, the most abundant miRNA in the liver, plays a fundamental role in hepatic lipid metabolism (Esau et al. 2006). miR-155 expression in NAFLD is dysregulated by the transcription factors C/EBP-α, C/EBP-β, PPAR-γ and LXRα (Virtue et al. 2017; Wang et al. 2016) and acts as a central node coordinating FGF21-mediated effects on liver metabolism (see below; Hochreuter et al. 2019). miR-143, which targets *Orp8*, is upregulated in the liver of obese mice and involved in the induction of insulin resistance (Jordan et al. 2011). Similarly miR-802 is upregulated liver of obese mice and human and its overexpression in mice caused insulin resistance, and its inhibition improved glucose tolerance. *Hnf1b* was identified as a target of miR-802, which itself improved insulin sensitivity when it was overexpressed in obese mice (Kornfeld et al. 2013). In addition, miR-103 and miR-107 were shown to be upregulated in obese mice and their silencing improved insulin sensitivity, presumably by affecting their target caveolin-1, a critical regulator of the insulin receptor (Trajkovski et al. 2011). miR33a/b interferes with insulin signaling and fatty acid metabolism by targeting *CROT*, *CPT1a*, *HADHB*, *AMPKα*, and *IRS2* (Davalos et al. 2011). miR-26a is downregulated in livers of overweight persons and leptin-deficient B6-ob/ob mice. Global and liver-specific transgenic miR-26a mice are protected from obesity-mediated side effects, show an improved insulin sensitivity, decreased hepatic glucose production, and decreased fatty acid synthesis. Conversely, inhibition of miR-26a expression in mice resulted in opposite effects. Targets of miR-26a are key regulators of hepatic metabolism and insulin signaling (*Acsl3*, *Acsl4*, *Gsk3b*, *Pck1*) (Fu et al. 2015).

In livers from mouse models for NAFLD and of those from the same mice treated with FGF21 (mimicking fasting conditions and improved liver metabolism) two miRNA nodes, sharing the same targets, were identified to coordinate the regulation of hepatic energy metabolism and insulin sensitivity. One node of the two miRNAs, miR-883b and miR-205, was downregulated in hepatosteatosis resulting in an enhanced expression of the transcription factor *ZEB1*. A second node, the upregulation of miR-155 and miR-1968 in response to FGF21 treatment regulates hepatic energy metabolism by downregulating *PTRF*. *PTRF* is involved in the transcriptional regulation and essential for caveolae formation (Hochreuter et al. 2019). In detail, in fatty liver of HFD-fed mice 24 miRNAs showed an altered expression in comparison to lean controls, whereas 338 were affected in liver-specific insulin receptor KO (LIRKO) mice, indicating that the insulin receptor signaling mediates stronger effects. Combining both NAFLD models resulted in 16 miRNAs with expression changes in the same direction. Only two of them (miR-802 and miR-107) were already linked to hepatic insulin resistance. Nineteen miRNAs with an opposite regulation were detected after FGF21 treatment (Hochreuter et al. 2019).

## miRNA in circulation

miRNA can be packaged into exosomes and secreted from cells. Recently, several groups showed the implications of the exosomal miRNA in obesity-related traits (Ying et al. 2017; Castano et al. 2018; Thomou et al. 2017). Exosomal miRNAs are released from multiple tissues but adipose tissue was the most studied in this context. By deleting the miRNA-processing enzyme Dicer in adipocytes (ADicerKO mice), Thomou and colleagues demonstrated that adipose tissue is a major source of exosomal miRNA (Thomou et al. 2017). This finding was also verified in humans with lipodystrophy, a disease characterized with low expression of Dicer. The transplantation of white and especially brown adipose tissue (BAT) into ADicerKO mice restored circulating of miRNAs and improved glucose tolerance. Ying and colleagues focused in exosomal miRNAs released from adipose tissue macrophages (ATMs). They demonstrated that ATMs secrete exosomal miRNAs, and that those released from ATMs of obese mice are sufficient to mediate glucose intolerance and insulin resistance. miR-155 for example is higher expressed and released by obese ATMs and targets *PPARγ*. The important role of miR-155 was shown by the phenotype of the corresponding knockout mouse. It exhibits an improved insulin sensitivity and glucose tolerance in comparison to wild-type mice. The bone marrow transplantation of wild-type mice into miR-155-KO mice attenuated this effect indicating that secreted miR-155 can reach insulin responsive cells and presumably acts via paracrine or endocrine regulation on glucose homeostasis (Ying et al. 2017).

Castaño et al. examined the global changes in plasma exosomal miRNA profiles in obese mice. Obesity was linked to increased levels of exosomal miR-27a, -27b, 122, and -192 in plasma. Treating lean mice with exosomes isolated from obese mice induces glucose intolerance and insulin resistance. In addition, injection of control exosomes containing obesity-associated miRNA mimics to lean mice induced glucose intolerance, obesity and hepatic steatosis (Castano et al. 2018).

Circulating miRNAs are not always packed in exosomes. Arroyo et al. demonstrated that protein complexes protect circulating miRNAs from plasma RNases. After its synthesis in the cells, mature miRNAs associate with Argonaute2 (Ago2) which mediates messenger RNA silencing activity (Kim et al. 2009). Interestingly, Ago2 was present in human plasma and non-vesicle-associated plasma miRNAs were detected in immunoprecipitation complexes of Ago2. The analysis of Arroyo and colleagues demonstrated that the larger proportion of miRNAs in human plasma associates with the Ago2 ribonucleoprotein complex, whereas a smaller part is present in exosomes (Arroyo et al. 2011). Due to the stability of circulating miRNAs, global profiling of miRNAs in plasma of lean and obese persons was already investigated. Two groups found miRNAs in the circulation which were already described to play a role in obesity like miR-28, -32, -122, -192, -374b, and -378a. Let7b, miR-34a, -144, and -532 were significantly correlated with clinical parameters related to insulin resistance (Jones et al. 2017; Wang et al. 2015). Taken together, as circulating miRNAs are stable because of their association to proteins or their localization in exposomes, they play a more and more important role as blood-based biomarkers for metabolic diseases including obesity (Fig. 3).

## Conclusion

Obesity is a complex disease with a large interindividual heterogeneity which is in part caused by epigenetic differences. We are still in the beginning collecting data on DNA methylation patterns and miRNA expression profiles and understanding the epigenetic contribution to the development of overweight. Up to now, at least in most human studies only data on DNA methylation or miRNA levels in blood cells are described without information on their effects on mRNA expression. Therefore, a clear and reliable conclusion about their impact on gene expression in specific tissues, on pathways and effects on body weight development can only be speculated, and detailed studies in animal models and analysis in human tissue biopsies are needed. In summary, literature presented in the current review indicates that the majority of epigenetic changes appear to be established during early development. However, it is still unclear whether these early changes are stable or susceptible to variation. Longitudinal studies are mandatory to answer the question to which extent and by which intervention or treatment epigenetic modifications are reversible. In addition, in the future, results of epigenetic studies will be used for stratification of people at risk for obesity and obesity-related diseases and for prediction of the success of specific treatments. Knowledge on the epigenome might facilitate the definition of subgroups exhibiting a specific set of plasma miRNAs and/or a specific DNA methylation pattern for which other studies have shown that for instance exercise changes this pattern. Other subgroups might carry other epigenetic marks which are affected by specific diets, intermittent fasting or other interventions. For these future directions we need more studies (i) on larger well-characterized cohorts, (ii) novel tools to detect all and the most relevant epigenetic changes, (iii) on appropriate animal models reflecting the human pathophysiology, (iv) investigating which epigenetic alterations are reversible by which intervention, (v) a long follow-up on intervention studies, and (vi) intensive bioinformatic analysis combining the different information.
